# Safety of bevacizumab and olaparib as frontline maintenance therapy in advanced ovarian cancer: expert review for clinical practice

**DOI:** 10.3389/fonc.2023.1304303

**Published:** 2024-01-29

**Authors:** Ignacio Romero, Eva Guerra, Ainhoa Madariaga, Luis Manso

**Affiliations:** ^1^ Department of Medical Oncology, Instituto Valenciano de Oncología (IVO), Valencia, Spain; ^2^ Department of Medical Oncology, Ramón y Cajal University Hospital, Madrid, Spain; ^3^ Department of Medical Oncology, 12 de Octubre University Hospital, Madrid, Spain

**Keywords:** ovarian cancer, olaparib, bevacizumab, maintenance therapy, first-line, toxicity management

## Abstract

Olaparib, a poly(ADP-ribose) polymerase inhibitor, in combination with the antiangiogenic agent bevacizumab, is approved as maintenance therapy for patients with newly diagnosed stage III or IV epithelial ovarian cancer who have homologous recombination deficient tumors with a deleterious or suspected deleterious *BRCA* mutation and/or genomic instability based on the long-lasting survival benefit observed in the PAOLA-1 trial. Despite treatment with olaparib and bevacizumab showing an acceptable safety profile, the rate of discontinuations due to adverse events was relatively high, and toxicity related to this regimen may restrict its clinical use. Proper management of olaparib/bevacizumab-related adverse events is important for the improvement of quality of life and maximization of the efficacy of maintenance therapy. Here, we summarize the safety results of the PAOLA-1 study, focusing on treatment discontinuation reasons and adverse event profiles. We sought to shed light on toxicity monitoring and prevention, providing concise recommendations for the clinical management of the most relevant side effects.

## 1 Introduction

Epithelial ovarian cancer (EOC) continues to be the most lethal gynecological tumor. Diagnosis is usually made at advanced stages, and cytoreductive surgery and first-line platinum-based chemotherapy have been the standard of care for decades. However, the majority of patients relapse within 3 years, with no reliable biomarkers to timely detect disease recurrence (1, 2). Two pivotal trials, GOG-0218 (3) and ICON7 (4), confirmed in 2011 the benefit of continuation maintenance therapy with bevacizumab, a humanized monoclonal antibody against vascular endothelial growth factor (VEGF), leading to its approval in the USA, the European Union, and other countries worldwide to treat patients with FIGO (International Federation of Gynecology and Obstetrics) stage III or IV EOC following debulking surgery (5).

Genome instability is a hallmark of EOC. In 50% of high-grade serous epithelial carcinoma, the most common histological subtype, there is a homologous recombination deficiency (HRD) due to different mechanisms, with mutations in *BRCA1/2* (14.5% germline and 7% somatic mutations) the most prevalent (6). Based on the molecular mechanisms of actions that target key DNA repair pathways in cancer cells, poly(ADP-ribose) polymerase inhibitors (PARPis) have emerged as a new therapeutic option in the management of EOC, particularly for tumors presenting HRD (7, 8). Currently, there are three PARPis in EOC approved by U.S. Food and Drug Administration (FDA) and European Medicines Agency (EMA)—olaparib, niraparib, and rucaparib—with different clinical indications and toxicity profiles (**Table 1**).

**Table 1:** Poly(ADP-ribose) polymerase inhibitor (PARPi) approved indications in ovarian cancer (updated to June 2023).

Drug	FDA-approved indications	EMA-approved indications
Olaparib	• Maintenance treatment of adult patients with deleterious or suspected deleterious germline or somatic *BRCA*-mutated advanced EOC, FTC, or PPC who are in CR or PR to first-line platinum-based chemotherapy • In combination with bevacizumab, for the maintenance treatment of adult patients with advanced EOC, FTC, or PPC who are in CR or PR to first-line platinum-based chemotherapy and whose cancer is associated with HRD-positive status defined by either a deleterious or suspected deleterious *BRCA* mutation and/or genomic instability • Maintenance of recurrent EOC, FTC, or PPC in patients with CR or PR to platinum-based chemotherapy, regardless of *BRCA* status	• Maintenance treatment of adult patients with advanced (FIGO stages III and IV) *BRCA1/2*-mutated (germline and/or somatic) high-grade EOC, FTC, or PPC who are in response (CR or PR) following completion of first-line platinum-based chemotherapy • Maintenance treatment of adult patients with platinum-sensitive relapsed high-grade EOC, FTC, or PPC who are in response (CR or PR) to platinum-based chemotherapy • In combination with bevacizumab, for the maintenance treatment of adult patients with advanced high-grade EOC, FTC, or PPC who are in response (CR or PR) following completion of first-line platinum-based chemotherapy in combination with bevacizumab and whose cancer is associated with HRD positive status defined by either a *BRCA1/2* mutation or genomic instability
Niraparib	• Maintenance treatment of adult patients with advanced EOC, FTC, or PPC who are in a CR or PR to first-line platinum-based chemotherapy • Maintenance treatment of adult patients with deleterious or suspected deleterious gBRCAm recurrent EOC, FTC, or PPC who are in a CR or PR to platinum-based chemotherapy	• Maintenance treatment of adult patients with advanced EOC, FTC, or PPC who are in response (CR or PR) following completion of first-line platinum-based chemotherapy • Maintenance treatment of adult patients with platinum-sensitive relapsed high-grade serous EOC, FTC, or PPC who are in response (CR or PR) to platinum-based chemotherapy
Rucaparib	• Treatment of adult patients with deleterious *BRCA* mutation (germline and/or somatic)-associated EOC, FTC, or PPC who are in a CR or PR to platinum-based chemotherapy	• Maintenance treatment of adult patients with platinum-sensitive relapsed high-grade serous EOC, FTC, or PPC who are in response (CR or PR) to platinum-based chemotherapy

CR, complete response; EOC, epithelial ovarian cancer; EMA, European Medicines Agency; FDA, U.S. Food and Drug Administration; FIGO, International Federation of Gynecology and Obstetrics; FTC, fallopian tube cancer; gBRCAm, germline BRCA-mutated; HRD, homologous recombination deficiency; PPC, primary peritoneal cancer; PR, partial response.

In the frontline maintenance setting, olaparib was the first PARPi to be approved for the treatment of *BRCA* mutated FIGO stage III or IV EOC, as a switch maintenance strategy, based on the SOLO-1 (NCT01844986) study results (9). Niraparib was approved with the same indication regardless of the biomarker status, following PRIMA (NCT02655016) study results (10). Both are approved for patients who have complete or partial response upon completion of first-line platinum-based chemotherapy. In this setting, although not yet granted regulatory approval, rucaparib maintenance was also associated with longer progression-free survival (PFS) (11). An additional PARPi, veliparib, not yet approved but has been added to first-line chemotherapy and continued as maintenance monotherapy, has been shown to improve the PFS of EOC patients, particularly those with HRD (12). With a different approach, olaparib is the only PARPi approved in combination with bevacizumab for newly diagnosed stage III or IV EOC with HRD tumors harboring deleterious or suspected deleterious *BRCA* mutations and/or genomic instability based on the benefit observed in the PAOLA-1 study (13).

Preclinical studies suggest that PARPis and antiangiogenic combinations may provide enhanced benefits in EOC (14, 15). Exploratory analyses point out that the combination of olaparib and bevacizumab may derive higher improvement in PFS compared to monotherapy (16, 17). Despite olaparib and bevacizumab showing acceptable safety profiles, up to 20% of participants in the PAOLA-1 trial discontinued due to adverse events (AEs) (13). Thus, toxicity related to this regimen and its management may raise concerns about the use of the combination in clinical practice. The aim of this review was to examine safety data from the PAOLA-1 study, focusing on treatment discontinuation reasons and the AE profile. In addition, we sought to shed light on toxicity monitoring and management to optimize the integration of this maintenance regimen in clinical practice. Management recommendations for treatment-emergent AEs (TEAEs) are proposed based on trial protocol, prescribing information, published supportive cancer care guidelines, and the authors’ clinical experience.

## 2 Delving into the PAOLA-1 study

PAOLA-1 was a randomized, double-blind study that compared olaparib (300 mg, twice daily for up to 24 months) with bevacizumab (15 mg/kg every 3 weeks for up to 15 months) as maintenance therapy after first-line chemotherapy in patients with newly diagnosed, advanced FIGO stage EOC, regardless of *BRCA* mutation status and surgical outcomes, who were in complete or partial response to standard first-line platinum–taxane-based chemotherapy and bevacizumab (13). As the primary endpoint, time from randomization to investigator-assessed disease progression or death was chosen. After a median follow-up of 22.9 months, significant increases in PFS were observed for olaparib plus bevacizumab compared with placebo plus bevacizumab in the overall population (median PFS 22.1 *vs.* 16.6 months; hazard ratio, 0.59; 95% confidence interval [CI], 0.49 to 0.72; p < 0.001), with the greatest PFS benefit seen in patients with *BRCA* mutations (37.2 *vs.* 21.7 months) and those with HRD-positive status, regardless of *BRCA* mutation status (37.2 *vs.* 17.7 months). In patients with HRD-positive tumors without *BRCA* mutations, the median PFS increased to 28.1 months for olaparib compared to 16.6 months in the placebo group (13). Significant increases were also observed for time to second objective disease progression (PFS2) (36.5 months for olaparib plus bevacizumab *vs.* 32.6 months for placebo plus bevacizumab; hazard ratio, 0.78; 95% CI, 0.64 to 0.95; p = 0.0125). Median time to second subsequent therapy and death (TSST) was 38.2 months and 31.5 months, respectively (hazard ratio, 0.78; 95% CI, 0.64 to 0.95; p = 0.0115). In the placebo plus bevacizumab group, 72 (27%) patients received a PARPi as the first subsequent therapy (18). The final analysis of the overall survival (OS) at 5 years has shown that, in HRD-positive patients, olaparib plus bevacizumab reduced the mortality risk by 38% versus bevacizumab, and 65.5% of patients treated with the combination were still alive at 5 years compared to 48.4% of those treated with bevacizumab alone (19). These numbers of long survivors are similar to those reflected by the OS rates at 7 years of follow-up in the SOLO-1 trial (67.0% of olaparib patients *vs.* 46.5% of placebo patients) (20), which support the potential for cure of maintenance olaparib.

**Figure 1:**
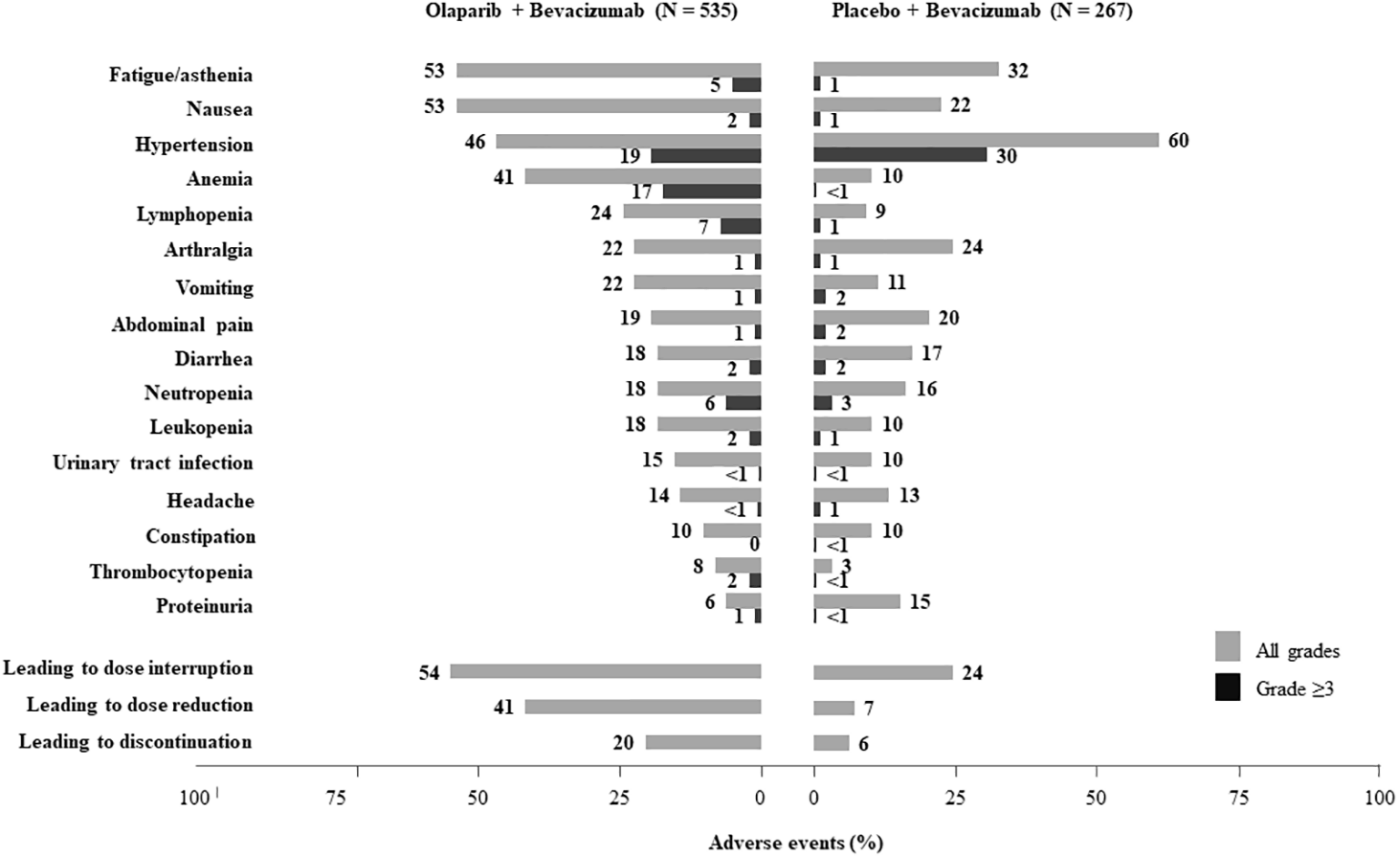
Summary of adverse events in the PAOLA-1 trial (13).

In the PAOLA-1 trial, more patients in the olaparib plus bevacizumab group (148/535 [27.7%] patients) completed the per protocol maximum 2-year treatment period than those in the placebo plus bevacizumab group (53/267 [19.9%] patients). The main toxicities reported in the PAOLA-1 study are summarized in **Figure 1**. Overall, the most common TEAEs ( ≥ 20%) associated with olaparib and bevacizumab versus bevacizumab and placebo were fatigue/asthenia (53% *vs.* 32%, respectively), nausea (53% *vs.* 22%), hypertension (46% *vs.* 60%), anemia (41% *vs.* 10%), lymphopenia (24% *vs.* 9%), vomiting (22% *vs.* 11%), and arthralgia (22% *vs.* 24%). The most relevant grade 3–4 toxicities (≥ 5%) with olaparib and bevacizumab compared to bevacizumab and placebo were hypertension (19% *vs.* 30%), anemia (17% *vs.* < 1%), lymphopenia (7% *vs.* 1%), fatigue/asthenia (5% *vs.* 1%), and neutropenia (6% *vs.* 3%).

The combined olaparib and bevacizumab regimen led to dose interruptions in 54% of patients, reductions in 41%, and discontinuations in 20%, compared to bevacizumab and placebo, which caused 24%, 7%, and 6% interruption, reduction, and discontinuation, respectively. Of note, patients who discontinued study treatment in PAOLA-1 were proactively questioned if this was due to an AE, whereas this approach was not taken in other trials (9–11). This difference in managing discontinuations may have contributed to the high rate of discontinuation due to AEs observed in this study. In fact, the overall discontinuation rates due to TEAEs, patient decision, and other reasons were comparable in the PAOLA-1 (25% of patients in the olaparib plus bevacizumab arm) (13), SOLO-1 (24% of patients in the olaparib arm) (9), PRIMA (18% of patients in the niraparib arm) (10), and ATHENA-MONO (18% of patients in the rucaparib arm) (11) studies (**Table 2**). The tolerability profile of olaparib plus bevacizumab versus placebo plus bevacizumab was consistent across the higher and lower risk of progression subgroups of patients and similar regardless of biomarker status (21). There was no clinically significant change in health-related quality of life in either group and no relevant difference between the treatment groups (13).

**Table 2:** Discontinuation rates in poly(ADP-ribose) polymerase inhibitor (PARPi) clinical trials.

Reasons for discontinuation other than disease progression, n (%)	PAOLA-1 (13)		SOLO-1 (9)		PRIMA (10)			ATHENA-MONO (11)	
	Olaparib (n = 535)	Placebo (n = 269)	Olaparib (n = 260)	Placebo (n = 130)	Niraparib, fixed dose (n = 317)	Niraparib, individualized dose (n = 170)	Placebo (n = 244)	Rucaparib (n = 425)	Placebo (n = 110)
TEAE	109 (20)	13 (5)	30 (12)	3 (2)	35 (11)	23 (14)	5 (2)	50 (12)	6 (5)
Patient decision	4 (1)	4 (2)	22 (8)	2 (2)	10 (3)	2 (1)	1 (0)	21 (5)	3 (3)
Other	19 (4)	6 (2)	11 (4)	9 (7)	14 (4)	4 (2)	7 (3)	7 (1)	2 (2)
**Total**	**132 (25)**	**23 (9)**	**63 (24)**	**14 (11)**	**59 (19)**	**29 (17)**	**13 (5)**	**78 (18)**	**11 (10)**

TEAE, treatment-emergent adverse event.

Toxicities observed during treatment with olaparib and bevacizumab were mostly managed by dose reductions and interruptions. Olaparib dose reductions were scarcely required, and AEs were managed by dose interruptions in most patients. Dose reductions occurred mainly during the first 3 months of therapy, in parallel with the temporal onset of the most common AEs (22). Prompt identification of any toxicity is mandatory, aiming to continue the treatment to optimize clinical benefit. If dose reductions are needed despite preventive/supportive measures, they should be implemented decreasing to 250 mg twice/day as a first step and further decreasing to 200 mg twice/day as a second step. Preliminary data indicate that there is a significant relationship between plasma olaparib exposure and the occurrence of serious AE (SAE). A trough plasma concentration threshold >2,500 ng/mL may be associated with a higher risk of SAE, which could guide dose adjustments in certain patients (23). Anticipatory and effective supportive care is critical to avoid dose changes. In the following sections, we propose practical guidance for the management of the most relevant AEs.

## 3 Management of adverse events in clinical practice (beyond drug label information)

### 3.1 Hematologic toxicity

#### 3.1.1 Anemia

In the PAOLA-1 study, more than half of anemia events were grade 1 or 2 (**Figure 1**), and anemia rarely leads to permanent discontinuation of treatment. According to the EMA’s Variation Assessment Report of Lynparza (22), olaparib dose was reduced in 99 (18.5%) patients, interrupted in 110 (20.6%), and permanently discontinued in 19 (3.6%). Anemia started early, generally within the first 3 months of olaparib initiation (median time to first event was 1.54 months), with no evidence of cumulative effect, as the risk of developing anemia remained quite constant over the entire exposure period. The majority (209/219 patients) of the first events of anemia with olaparib/bevacizumab were controlled satisfactorily (median time to resolution 1.41 months). Blood transfusions were required by 94 (17.6%) patients, and 26 (4.9%) of them needed more than one transfusion after starting study treatment. Most of the transfusions took place during the first 4 months of treatment. Thirty (5.6%) patients treated in the olaparib/bevacizumab arm received an erythropoiesis-stimulating agent (22).

Thus, frequent monitoring for hematologic toxicity is recommended at the beginning of olaparib/bevacizumab maintenance therapy. A hemogram should be performed monthly for the first 12 months and periodically after this time to monitor for clinically significant changes in any parameter during treatment. If anemia appears, it is also necessary to study other possible causes, and monitoring of iron, folic acid, and vitamin B_12_ levels is recommended. Folate deficiency has been observed in some patients receiving olaparib, so administering folate supplements and/or other hematinics could ameliorate the risk of severe anemia in these cases (24). When hemoglobin values fall below 8 g/dL, olaparib dose reduction and/or blood transfusion should be considered (**Table 3**). If anemia persists below 8 g/dL for more than 4 weeks, refer the patient to hematology before continuing treatment and recommend bone marrow and/or blood cytogenetic analysis. Given that bevacizumab may increase the risk of bleeding, the presence of active bleeding should be checked. If the hemoglobin falls back to less than 8 g/dL after dose reductions or there is a need for periodic transfusions, then definitive discontinuation of olaparib treatment is recommended.

**Table 3:** Proposal for anemia management.

Severity	Recommended action
CTCAE grade 2 (Hb < 10.0 to ≥ 8.0 g/dL)	First event - Provide supportive treatment and investigate causality - According to physician’s judgment, continue olaparib or interrupt dose for a maximum of 4 weeks
	Recurrent event - Interrupt olaparib for a maximum of 4 weeks until Hb ≥ 10 g/dL. If more than 4 weeks is required, refer the patient to hematology service - When Hb ≥ 10 g/dL, resume olaparib with a reduced dose: • 250 mg twice daily as a first step and • 200 mg twice daily as a second step if it recurs
CTCAE grade 3 (Hb < 8.0 g/dL)	First event - Interrupt olaparib for a maximum of 4 weeks until Hb ≥ 10 g/dL. If more than 4 weeks is required, refer the patient to hematology service - When Hb ≥ 10 g/dL, resume olaparib with the first step of reduced dose (250 mg twice daily)
	Recurrent event - Interrupt olaparib for a maximum of 4 weeks until Hb ≥ 10 g/dL with supportive treatment. If more than 4 weeks is required, refer the patient to hematology service - When Hb ≥ 10 g/dL, resume olaparib with the second step of reduced dose (200 mg twice daily)
CTCAE grade 3 (Hb < 8.0 g/dL) with concurrent neutropenia and/or thrombocytopenia	- Interrupt olaparib and bevacizumab for a maximum of 4 weeks until recovery with supportive treatment. If more than 4 weeks is required, refer the patient to hematology service - When Hb ≥ 10 g/dL, resume bevacizumab according to clinical practice and resume olaparib with the second step of reduced dose (200 mg twice daily)
CTCAE grade 3 (Hb < 8.0 g/dL) despite dose reduction or more than one transfusion is needed for anemia recovery	Definitely discontinue olaparib treatment

CTCAE, Common Terminology Criteria for Adverse Events; Hb, hemoglobin.

#### 3.1.2 Thrombocytopenia

In the PAOLA-1 study, thrombocytopenia or decreased platelet count of any grade was reported in 8% of patients in the olaparib plus bevacizumab arm (**Figure 1**), but no event led to treatment discontinuation. The development of thrombocytopenia was not associated with the duration of olaparib/bevacizumab treatment. Thrombocytopenia events appeared initially during the first 12 months of the study period in the olaparib/bevacizumab arm (median time to first onset, 1.41 months); most of the patients (42/43 patients) resolved satisfactorily (median time to resolution of the first event was 0.82 month) (22). Regarding hemorrhage events, 52/535 (9.7%) patients treated with olaparib plus bevacizumab had a total of 65 AEs, whereas in the placebo/bevacizumab arm, only 36 AEs were observed in 28/267 (10.5%) patients. Bleeding events were predominantly grade 1 or 2. Similar proportions of patients in each group (5/43 [11.6%] olaparib/bevacizumab and 1/9 [11.1%] placebo/bevacizumab) received treatment for thrombocytopenia AEs. Five (0.9%) versus one (0.4%) patients received platelet transfusions (22).

Although it seems that olaparib-associated thrombocytopenia rarely translates into bleeding risk, grade 1 thrombocytopenia (platelets <75,000/mm^3^) requires close monitoring and possible dose reduction (**Table 4**). If grade 2 or higher thrombocytopenia occurs, olaparib should be interrupted, and weekly monitoring should be carried out until recovery of platelets to the level of 100,000/mm^3^. Platelet transfusions are recommended when platelet counts are below 20,000/mm^3^, or higher with active bleeding or planned invasive procedure, or in patients with ulcerative tumors. In addition, patients who receive anticoagulants or antiplatelet therapy should also be considered for transfusion at higher platelet counts or when anticoagulation is interrupted (25).

**Table 4:** Recommendations for managing the main adverse events associated with the combination of olaparib and bevacizumab.

Adverse event		Proposed management
Hematologic toxicity	• Hemogram should be performed twice weekly for the first 2 months and monthly thereafter	
	Anemia	• Investigate other possible causes and monitor iron, folic acid, and vitamin B_12_ levels • Provide folate supplements and/or other hematinics, if required • Investigate the presence of active bleeding • If hemoglobin falls < 8 g/dL, follow the actions described in **Table 3**
	Thrombocytopenia	• Grade 1: Close monitoring and potential dose reduction • Grade ≥ 2: Interrupt olaparib and monitor weekly until recovery (100,000 platelets/mm^3^) • Consider platelet transfusion when platelets fall below 20,000/mm^3^, or higher with active bleeding, planned surgery, or ulcerative tumors • Be aware of concomitant anticoagulant or antiplatelet therapy
Non-hematologic toxicity	Nausea and vomiting	• Advise patients regarding these side effects • Dietary modifications: protein-rich foods, taking olaparib after meals • Consider antiemetic prophylaxis
	Fatigue/asthenia	• Inform patients of the expected patterns of fatigue • Recommend regular exercise, massage therapy, and/or psychosocial interventions • Consider vitamin B supplementation • If grade ≥ 3 fatigue, consider dose reductions or interruption
	Arthralgia	• Inform patients regarding this side effect • Consider referral to rheumatology • For mild pain, treat with common analgesics
	Hypertension	• Check and control pre-existing hypertension before initiating bevacizumab therapy • Measure blood pressure before and after the first few bevacizumab infusions and then every 3 weeks • In patients with a blood pressure of 150/100 mmHg or more, interrupt bevacizumab until normal pressure is restored using antihypertensive medication • When hypertension persists, refer the patient to specialized hypertension units or the general practitioner for adequate monitoring and follow-up
	Proteinuria	• Regular urine assessment • If abnormal urine results, perform 24-hour urine analysis • If proteinuria ≥ 2 g/24 hours, interrupt bevacizumab until recovery to < 2 g/24 hours

#### 3.1.3 Myelodysplastic syndrome/acute myeloid leukemia

Nine (1.7%) versus six (2.2%) cases of myelodysplastic syndrome/acute myeloid leukemia (MDS/AML) were reported in the olaparib versus placebo groups in the PAOLA-1 study, respectively (17). MDS/AML is considered an important identified risk of olaparib treatment. Warnings regarding blood count monitoring and discontinuation of treatment in the event of MDS/AML are included in the product information (26).

### 3.2 Non-hematologic toxicity

#### 3.2.1 Nausea and vomiting

In the PAOLA-1 study, most nausea and vomiting events were grade 1–2 (**Figure 1**), and most did not require treatment discontinuation (22). These gastrointestinal toxicities generally occurred early during treatment (median time to onset of nausea and vomiting were 0.16 and 1.38 months, respectively), and most events with olaparib/bevacizumab subsequently improved or resolved (median time to resolution of the first event of 1.28 and 0.10 months, respectively). Nausea prevalence decreased from a range of 0.3%–0.2% in the first 6 months of treatment to 0.2%–0.1% thereafter. Vomiting prevalence was approximately 0.05% throughout the study. A total of 104 (19.4%) patients in the olaparib plus bevacizumab arm reported both nausea and vomiting. In the olaparib plus bevacizumab treatment arm, approximately half of the patients with nausea (158/285 [55.4%] patients) required treatment, and 58/117 (49.6%) patients who experienced vomiting received treatment. Fewer patients received treatment for nausea and vomiting in the placebo plus bevacizumab group (24/58 [41.4%] and 10/29 [34.5%] patients, respectively). The incidence of nausea and vomiting was higher (≥5 percentage points difference) in patients aged <65 years when compared with older patients (22).

Patient advice regarding these side effects can help them be prepared and thereby continue with the treatment (**Table 4**). Eating bland foods and liquids that are easy on the stomach and consuming protein-rich foods are common dietary changes recommended by nutritionists (27). Taking olaparib tablets after breakfast and dinner can also be helpful. In patients with recurrent emesis, a prophylactic approach with antiemetic therapy (e.g., metoclopramide) may avoid dose adjustments or discontinuation (28).

#### 3.2.2 Fatigue/asthenia

More than half of patients on olaparib/bevacizumab treatment experienced fatigue or asthenia, most of the time graded 1 or 2 (**Figure 1**). Up to 28/535 (5.2%) patients had grade 3 fatigue (i.e., interfering with activities of daily living and self-care). These AEs were reported early, as most of the first events in the olaparib/bevacizumab arm were reported within the first 3 months of treatment. The median time to onset was 0.72 months, and the incidence plateaued at approximately 1 month (20). The majority (220/283 [77.7%] patients) of fatigue and asthenia events with olaparib/bevacizumab resolved in a median time of 2.10 months. Few patients in the olaparib/bevacizumab arm experiencing fatigue and asthenia (6/283 [2.1%] patients) required treatment compared to 3/86 (3.5%) in the placebo/bevacizumab group (22).

Although treatment-related fatigue represents a common class effect of PARPis, some subjective and objective underlying causes not directly related to these drugs could also be contributing to fatigue, such as prior chemotherapy, anemia, poor nutrition, emotional distress, or insomnia (**Table 4**) (29). Fatigue is a distressing symptom that negatively impacts patients’ quality of life and can provoke a lack of adherence (30). Patients should be informed of the expected patterns of fatigue, aiming to facilitate patients’ adaptation to the ongoing treatment. Regular exercise (for example, Pilates) may be recommended, along with massage therapy and/or psychosocial interventions (31). Vitamin B supplementation seems to improve anemia and fatigue and may be considered in selected cases (32). For patients with grade ≥ 3 fatigue, intolerable or long-lasting low-grade fatigue, dose reductions, or interruption may be necessary.

#### 3.2.3 Arthralgia

All grade arthralgia afflicted 116 (22%) patients of the olaparib plus bevacizumab group and 64 (24%) patients of the placebo plus bevacizumab group (**Figure 1**). In other studies, the incidence of arthralgia in bevacizumab-treated patients was higher, reaching up to 50% (33–35), with evidence of cumulative toxic effect (36). Health professionals should be aware of this side effect to inform patients and if needed refer them to a rheumatologist to properly manage joint pain. Treatment for mild pain may include common analgesics, but severe pain could require corticosteroids or methotrexate (**Table 4**) (35).

#### 3.2.4 Hypertension

Hypertension events were reported in a lower percentage of patients in the olaparib/bevacizumab arm (45.8%) when compared to the placebo/bevacizumab arm (59.9%). Fewer patients presented with grade 3 hypertension in the olaparib/bevacizumab group, compared with the placebo/bevacizumab group of the PAOLA-1 study (**Figure 1**). The majority of first hypertension events in both treatment arms occurred during the first 12 months of treatment. It was suggested that hypertension AEs were associated with bevacizumab treatment, as these events occurred at the same time of bevacizumab exposure (median duration of bevacizumab treatment, 11.0 months with olaparib/bevacizumab and 10.4 months with placebo/bevacizumab). In the first month of treatment, similar numbers of patients in each arm experienced a first hypertension AE, with rates of 17.8% for olaparib/bevacizumab and 20.2% for placebo/bevacizumab. Hypertension in the study rarely resulted in dose changes for olaparib or placebo, and none resulted in discontinuation (22).

Patients with pre-existing hypertension should have adequate blood pressure control prior to initiation of bevacizumab therapy. All patients should have blood pressure monitoring before and after the first few bevacizumab infusions and then every 3 weeks. In patients with a blood pressure of 150/100 mm Hg or higher, bevacizumab should be interrupted until normal blood pressure is restored with antihypertensive medications. If hypertension persists, the patient should be referred to specialized hypertension units or the general practitioner for adequate monitoring and follow-up (**Table 4**) (37, 38).

#### 3.2.5 Proteinuria

Proteinuria is one of the commonly reported side effects caused by bevacizumab, especially in patients with a history of hypertension (39). In the PAOLA-1 study, proteinuria was reported in more patients in the placebo/bevacizumab group (15.4%) than in the olaparib/bevacizumab one (5.8%) (**Figure 1**). Most proteinuria events were grade 1 or 2, and none led to treatment withdrawal. The majority of proteinuria AEs were observed during the combined treatment period in both groups (26/31 [83.9%] patients in the olaparib/bevacizumab arm and 37/41 [90.2%] patients in the placebo/bevacizumab arm). Similar onset times of proteinuria were observed in both arms. Most first-time proteinuria events occurred in the first 450 days of the study, which is consistent with an association with bevacizumab treatment (22).

Patients being treated with bevacizumab require regular systematic urine assessment. If any alteration is detected (two consecutive positive dipstick tests), a 24-hour urine analysis should be performed. In the event of urine proteinuria being greater than 2 g/24 hours, bevacizumab should be interrupted until recovery to <2 g/24 hours (**Table 4**) (40).

## 4 Concluding remarks and summary of recommendations

In summary, the PAOLA-1 study confirmed that adding olaparib to bevacizumab as maintenance therapy is beneficial in the group of patients who have HRD-positive tumors harboring a deleterious or suspected deleterious *BRCA* mutation and/or genomic instability, with manageable toxicity profile and no deterioration of health-related quality of life. Although the majority of olaparib-related AEs were mild and there was no evidence of an increase in the known toxic effects associated with bevacizumab, as with the administration of any treatment, a full discussion of the benefits and risks of the combination should take place with the patient as part of the informed-consent process. In addition, clinicians should detect and adequately manage the side effects of olaparib treatment as early as possible to maximize the efficacy of maintenance therapy. Toxic effects, many of which appear to be class effects of PARPis, are mostly self‐limiting and can be managed with the use of preventive and supportive measures and, sometimes, with treatment interruptions and/or dose reductions. We provide the summary of recommendations in **Table 4**, hoping to contribute to better clinical management of the potential toxicity. Although we provide a comprehensive and practical synthesis of the safety results of the PAOLA-1 study, the main limitation in making recommendations is the lack of real-world evidence on the long-term use of olaparib plus bevacizumab. However, on the basis of the critical review of the (scant) literature and the experience of the authors, we consider that the benefits of olaparib plus bevacizumab therapy currently outweigh potential risks, and therefore, patients with *BRCA* mutation or genomic instability in whom bevacizumab is added to the combination with first-line chemotherapy should not be deprived of the benefit of continuing bevacizumab in maintenance with olaparib.

## Author contributions

IR: Writing – original draft, Writing – review & editing. EG: Writing – original draft, Writing – review & editing. AM: Writing – original draft, Writing – review & editing. LM: Writing – original draft, Writing – review & editing.
